# Host tracheal and intestinal microbiomes inhibit *Coccidioides* growth *in vitro*

**DOI:** 10.1128/spectrum.02978-23

**Published:** 2024-06-04

**Authors:** Susana Tejeda-Garibay, Lihong Zhao, Nicholas R. Hum, Maria Pimentel, Anh L. Diep, Beheshta Amiri, Suzanne S. Sindi, Dina R. Weilhammer, Gabriela G. Loots, Katrina K. Hoyer

**Affiliations:** 1 Quantitative and Systems Biology, Graduate Program, University of California, Merced, Merced, California, USA; 2 Biosciences and Biotechnology Division, Lawrence Livermore National Laboratories, Livermore, California, USA; 3 Department of Applied Mathematics, University of California, Merced, Merced, California, USA; 4 Health Sciences Research Institute, University of California, Merced, Merced, California, USA; 5 Department of Molecular and Cell Biology, School of Natural Sciences, University of California, Merced, Merced, California, USA; ^6^Department of Orthopaedic Surgery, Lawrence J. Ellison Musculoskeletal Research Center, University of California Davis Health, Sacramento, California, USA; Broad Institute, Cambridge, Massachusetts, USA

**Keywords:** *Coccidioides*, lung microbiota, antibiotics

## Abstract

Coccidioidomycosis, also known as Valley fever, is a disease caused by the fungal pathogen *Coccidioides*. Unfortunately, patients are often misdiagnosed with bacterial pneumonia, leading to inappropriate antibiotic treatment. The soil *Bacillus subtilis*-like species exhibits antagonistic properties against *Coccidioides in vitro*; however, the antagonistic capabilities of host microbiota against *Coccidioides* are unexplored. We sought to examine the potential of the tracheal and intestinal microbiomes to inhibit the growth of *Coccidioides in vitro*. We hypothesized that an uninterrupted lawn of microbiota obtained from antibiotic-free mice would inhibit the growth of *Coccidioides,* while partial *in vitro* depletion through antibiotic disk diffusion assays would allow a niche for fungal growth. We observed that the microbiota grown on 2×GYE (GYE) and Columbia colistin and nalidixic acid with 5% sheep’s blood agar inhibited the growth of *Coccidioides*, but microbiota grown on chocolate agar did not. Partial depletion of the microbiota through antibiotic disk diffusion revealed diminished inhibition and comparable growth of *Coccidioides* to controls. To characterize the bacteria grown and identify potential candidates contributing to the inhibition of *Coccidioides*, 16S rRNA sequencing was performed on tracheal and intestinal agar cultures and murine lung extracts. We found that the host bacteria likely responsible for this inhibition primarily included *Lactobacillus* and *Staphylococcus*. The results of this study demonstrate the potential of the host microbiota to inhibit the growth of *Coccidioides in vitro* and suggest that an altered microbiome through antibiotic treatment could negatively impact effective fungal clearance and allow a niche for fungal growth *in vivo*.

**IMPORTANCE:**

Coccidioidomycosis is caused by a fungal pathogen that invades the host lungs, causing respiratory distress. In 2019, 20,003 cases of Valley fever were reported to the CDC. However, this number likely vastly underrepresents the true number of Valley fever cases, as many go undetected due to poor testing strategies and a lack of diagnostic models. Valley fever is also often misdiagnosed as bacterial pneumonia, resulting in 60%–80% of patients being treated with antibiotics prior to an accurate diagnosis. Misdiagnosis contributes to a growing problem of antibiotic resistance and antibiotic-induced microbiome dysbiosis; the implications for disease outcomes are currently unknown. About 5%–10% of symptomatic Valley fever patients develop chronic pulmonary disease. Valley fever causes a significant financial burden and a reduced quality of life. Little is known regarding what factors contribute to the development of chronic infections and treatments for the disease are limited.

## INTRODUCTION

*Coccidioides immitis* and *Coccidioides posadasii* are soil fungi responsible for the disease coccidioidomycosis, also known as Valley fever. *Coccidioides* is endemic to hot, dry regions such as the southwestern United States, Central America, and South America (1, 2). The fungus grows in the soil as mycelia prior to disarticulating into the infectious arthroconidia spores. Upon aerosolization, spores are inhaled into the lungs where they become endosporulating spherules causing respiratory distress. About 60% of Valley fever cases remain asymptomatic, while 40% experience flu-like symptoms that mostly resolve on their own, and of these, 5%–10% of infections result in chronic pulmonary disease (1). The biological factors contributing to acute or chronic coccidioidomycosis have yet to be fully elucidated. In addition, the disease is often misdiagnosed as bacterial pneumonia, resulting in 60%–80% of these misdiagnosed patients being treated with several rounds of antibiotics prior to accurate diagnosis (3). This is due to poor testing strategies and contributes to a growing problem not only of antibiotic resistance but also antibiotic-induced microbiome dysbiosis that contributes to several chronic disorders such as inflammatory bowel disease, rheumatoid arthritis, asthma, and type 2 diabetes, to name a few (4–7). Antibiotic-induced dysbiosis correlates to a prevalence of pathogenic bacteria (8, 9). The use of antibiotics significantly shifts the lung microbiota repertoire resulting in less diversity and a higher abundance of resistant bacteria than in untreated lungs (10). Increased susceptibility and colonization with *Salmonella*, *Shigella flexneri*, and *Clostridium difficile* in germ-free mice are associated with antibiotic treatment (11–14). It is unknown if this shift in commensals correlates to a reduced ability to clear *Coccidioides* infection in coccidioidomycosis.

The microbiome utilizes multiple mechanisms of inhibition to protect against invading pathogens. Direct competition for host nutrients can inhibit pathogen colonization (15). However, to overcome competition, pathogens often use nutrients that are not preferred by resident gut bacteria. Host microbiomes may also produce factors to protect their host niche from other bacteria, viruses, and fungi. These indirect mechanisms of protection involve promoting factors that enhance the intestinal epithelial barrier or promote innate and adaptive immunity to inhibit pathogen colonization (15, 16). A soil *Bacillus subtilis-*like species displays antifungal activity against *Coccidioides* growth, with a clear zone of inhibition between fungi and bacteria when grown *in vitro* (17). Whether host commensal bacteria can also inhibit *Coccidioides* by direct or indirect mechanisms is unknown. Furthermore, it is unknown how antibiotic treatment resulting from misdiagnosis further affects the interrelationship between the host lung microbiome and the invading fungal pathogen.

The 2007 Human Microbiome Project did not initially include the lungs as a site of investigation as the lung was long thought to be sterile (18). Culture-dependent techniques posed a challenge in lung microbiome collection as microbial abundance is low compared to other sites of the body and only 1% of all bacteria are culturable in the laboratory (18, 19). 16S rRNA sequencing methods used to identify microbial communities in a healthy lung identified *Firmicutes*, *Bacteroidetes*, *Proteobacteria*, *Fusobacteria*, and *Actinobacteria* as the most prevalent families (18, 20). At the operational taxonomic unit level, *Prevotella*, *Veillonella*, and *Streptococcus* are routinely identified as prevalent residents of the lung (20). The lungs are part of the lower respiratory system along with the trachea and primary bronchi. The upper respiratory tract consists of the nose, mouth, sinuses, pharynx, and larynx. Among healthy individuals, the microbiomes of the upper and lower respiratory tracts are indistinguishable (21). Recent studies of COVID-19 and respiratory syncytial virus infections have explored differences between intestinal and respiratory microbiomes due to antibiotic treatment (22–25). Until recently, most infection microbiome studies have focused on the influence of intestinal dysbiosis on infection (26). It is unknown if the upper and lower respiratory tracts or the intestinal microbiome change with the infection of *Coccidioides* and influence *Coccidioides* growth. For the purposes of this study, we investigated the impact of cultured tracheal microbiota, which we considered to be representative of the lung microbiota, on *Coccidioides* growth *in vitro*.

## MATERIALS AND METHODS

### Mice

Six- to ten-week-old C57BL/6 male and female mice (JAX #000664, The Jackson Laboratories, Bar Harbor, ME, USA) were purchased or bred for experiments. Mice from multiple dams were used for experiments.

### Agar plates

2× glucose yeast extract (GYE) agar plates were made in accordance with the following recipe: 2% wt/vol glucose (Fisher Scientific), 1% wt/vol yeast extract (Fisher Scientific), and 1.5% bacteriological agar (VWR) in diH_2_O. GYE was autoclaved at 121°C for 1 h, poured into 100 × 15 mm^2^ petri dishes (Fisher Scientific), and stored at 4°C. Columbia colistin and nalidixic acid (CNA) agar with 5% sheep blood (5%SB-CNA) and chocolate agar plates were purchased from Fisher Scientific. 5% SB-CNA agar provides selective culture of Gram-positive bacteria, while chocolate agar is a fairly non-selective agar for fastidious Gram-negative bacteria growth.

### Arthroconidia harvest

NR-166 avirulent *Coccidioides posadasii* (Δ*cts2/*Δ*ard1/*Δ*cts3*) derived from *C. posadasii* strain C735 was used for all experiments (BEI Resources, Manassas, VA, USA) (27). Fungal glycerol stock was inoculated into liquid GYE media and cultured for 3–7 days at 30°C, 150 rpm in a shaking incubator. Liquid culture was streaked onto GYE agar plates and grown for 4–6 weeks to reach confluency and appropriate desiccation. To harvest arthroconidia, fungi were scraped off the plate using cell scrapers into a conical tube with phosphate-buffered saline (PBS). The collection was vortexed for 1 min prior to filtering through a 40 µm mesh filter to dislodge any arthroconidia withheld in the segmented mycelia encasing. Fungus was vortexed again for 1 min and washed twice with PBS (centrifuged at 12,000 × *g* for 8 min and then 20 min at room temperature with the break off). The fungal pellet was resuspended in PBS. Viability was assessed by plating 10-fold serial dilutions and colony counting 3–4 days post-plating. Arthroconidia suspension was stored at 4°C for up to 3 months. The complete protocol can be found in Mead et al. (28).

### Tracheal and intestinal microbiota growth

The trachea and small intestine were harvested under sterile conditions. The trachea was harvested by opening the chest cavity and cutting at the top of the bronchiole branching and base of the larynx. The trachea was cut in half, inverted onto a respective agar plate, and spread. About 3–4 cm of the small intestine closest to the stomach was harvested, cleaned of fecal material and major mucus contents, cut in half, and spread onto a respective agar plate. Plates were incubated for 48 h at 30°C–35°C. If 80% confluency was reached from direct plating, plates were used for spike-in inhibition assays. For the trachea, if ~80% confluency was not obtained from direct plating, then tracheal microbiota was harvested from the plate into 2 mL of PBS; serial dilutions were performed and plated for 48 h. The serial dilution from each trachea that yielded ~80% confluency was used for spike-in inhibition assays.

### 50/50 inhibition assay

Small intestine was harvested as described above and spread across half the GYE plate. Blank and PBS spread plates were used as controls. Simultaneously, 50 arthroconidia in 50 µL were spread across the other half of the GYE plate. Plates were incubated at 30°C–35°C for 11 days.

### Spike-in inhibition assay

Trachea and small intestine were harvested and spread across the entire agar plate. Blank and PBS spread plates were used as controls. Plates were incubated for 48 h at 30°C–35°C prior to spiking in 50 arthroconidia on the edge of the plate. Plates were incubated for an additional 11 days at 30°C–35°C.

### Disk diffusion spike-in inhibition assay

Trachea and small intestine were harvested and spread across GYE agar plates. Blank and PBS spread plates were used as controls. A volume of 100 µL of broad-spectrum antibiotic cocktail (ampicillin, rifampicin, streptomycin, and neomycin; 50 µg/mL each) or PBS control was placed onto a 2 cm diameter Whatman paper circle disk and placed in the center of the host microbiota spread for 48 h at 30°C–35°C. After 48 h, the disk was removed, and 50 arthroconidia were spiked onto the center of the plate. Plates were incubated for an additional 11 days at 30°C–35°C, with imaging at days 4, 7, and 11.

### Inhibition measurements

Pictures of agar plates were taken on days 4, 7, and 11, and the area of *Coccidioides* growth was measured using ImageJ software (Wayne Rasband and contributors, Version 1.53k). The scale was set to 8.5 cm for every agar plate prior to tracing the area of *Coccidioides* colony growth. The area was determined based on these measurements and recorded.

### Whole organ harvest for 16S rRNA sequencing

Replicates were included from different dams, and an equal number of female and male mice were used for 16S rRNA sequencing experiments. Right lung lobes were harvested and stored for bacterial extraction at −80°C.

### Bacterial DNA extraction

Tracheal and intestinal growth on agar plates were harvested using 1 mL of PBS via cell scraping and centrifuged for 10 min at 7,500 rpm. The microbial pellet was resuspended in 180 µL of enzymatic lysis buffer (20 mM Tris-Cl, pH 8, 2 mM sodium EDTA, 1.2% Triton X-100, and 20 mg/mL lysozyme added immediately before use). Harvested right lung lobes were cut into small pieces, and the microbial content of all plated and whole organ samples were isolated using the DNeasy Blood and Tissue Kit (Qiagen) following the manufacturer’s protocols for the extraction of bacterial content. DNA concentration was determined using NanoDrop.

### 16S rRNA sequencing

16S rRNA sequencing was utilized to identify the bacterial abundance and composition of microbiota derived from trachea and small intestine grown on different agar types and from right lung lobes. DNA extracts of bacterial samples were prepared according to the Illumina 16S Metagenomic Sequencing Library Preparation protocol. The Illumina protocol targeted variable 3 (V3) and V4 regions of the 16S ribosomal RNA gene for sequencing. PCR amplification of the target area was performed using the 2× KAPA HiFi Hot Start Ready Mix (070988935001, Roche). Reverse and forward amplicon PCR primers recommended by Illumina were used (16S Amplicon PCR Forward Primer: TCGTCGGCAGCGTCAGATGTGTATAAGAGACAGCCTACGGGNGGCWGCAG; 16S Amplicon PCR Reverse Primer: GTCTCGTGGGCTCGGAGATGTGTATAAGAGACAGGACTACHVGGGTATCTAATCC). After PCR amplification, the V3 and V4 amplicons were purified using AMPure XP beads (A63881, Beckman Coulter). To attach dual indexes and Illumina sequencing adapters, additional PCR amplification was conducted using the Nextera XT Index Kit (15032350, Illumina). The final library was purified once again using AMPure XP beads. Libraries were sequenced using the Illumina MiSeq sequencer (Illumina).

### 16S rRNA analysis

All analyses were performed using R version 4.1.3 with DADA2 version 1.22.0. Sequence reads were first pre-processed to trim off the primer sequence and truncated at 245 bp length for forward reads and at 179 bp length for reverse reads to facilitate the technical quality drop at the beginning of the forward reads and at the end of both forward and reverse reads, then processed through the DADA2 pipeline to identify amplicon sequence variants (ASVs) with chimeras being removed. These ASVs were classified to the genus level using the Ribosomal Database Project naive Bayesian classifier in combination with the SILVA reference database version 138.1 with minBoot = 50 (the minimum bootstrap confidence for assigning a taxonomic level).

Singletons were removed for all downstream analyses. We also removed ASVs with a phylum of NA and ASVs with ambiguous phylum annotation. Low-yield samples were not included in the downstream analysis (10–12 or 6 ng/µL for whole organ lung samples). A total of 332 ASVs were identified in 25 samples, with 19 plated organ samples, 2 negative control samples (pooled blank 5%SB-CNA, chocolate, and GYE plate samples), 3 whole organ samples, and 1 positive control sample (ATCC 10 strain control). The smallest number of reads per sample is 24,434 (the R lung lobes whole organ sample with ID 32). We further removed two plated trachea samples (IDs 5 and 20) as both their absolute abundance and relative abundance composition were significantly different from other samples in the same group (trachea samples plated on GYE plates for sample ID 5 and trachea samples plated on chocolate plates for sample ID 20). A total of 31 ASVs were identified in those two negative control samples, and these ASVs were removed from trachea and intestine-plated samples as well as the whole organ right lung lobe samples. The raw data of 16S rRNA sequencing were deposited in the Sequence Read Archive (SRA) at NCBI under BioProject PRJNA1081448.

### Statistics

Preliminary inhibition experiments were used to perform power calculations in G* Power; *t*-test, means: difference between two independent means (two groups), *a priori*: compute required sample size, two tails, power = 0.90, *α* = 0.05, to define replicate requirements.

Post-experimental statistics were performed using GraphPad Prism [version 10.1.1 (323)]. Two-way ANOVA statistical analysis was performed for the 50/50 inhibition assay, the intestine spike-in inhibition assay on 5%SB-CNA and chocolate agar, and the trachea spike-in inhibition assays on 5%SB-CNA and GYE agar data with Šídák corrections for multiple comparisons and a 95% confidence interval. Mixed effect analysis was performed for the intestine spike-in inhibition assays on GYE with Šídák corrections for multiple comparisons and a 95% confidence interval. An unpaired parametric *t*-test with Welch’s correction and a 95% confidence interval was performed on day 7 disk diffusion assay.

## RESULTS

**Fig 1:**
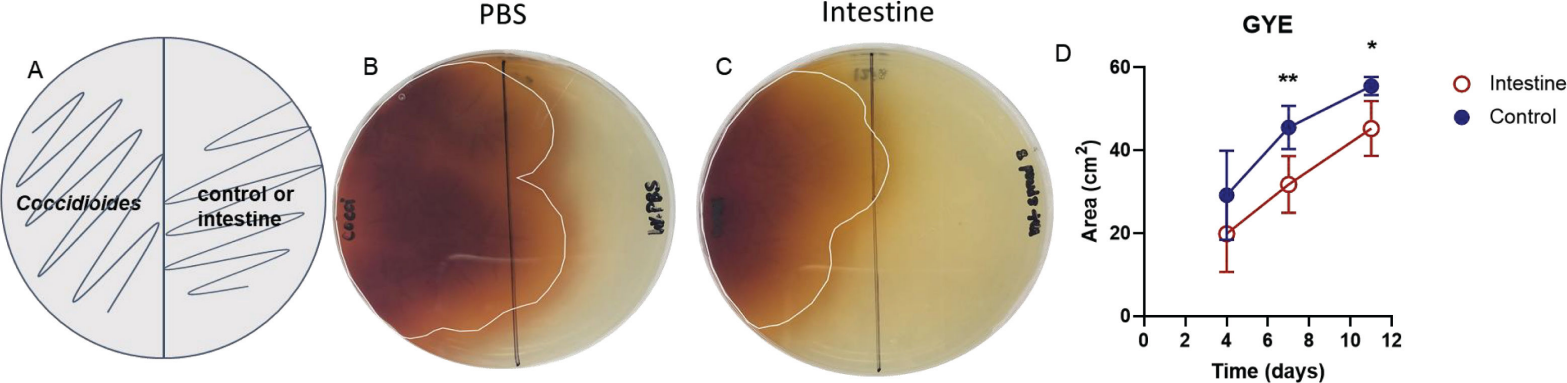
Intestinal mouse microbiota inhibits *Coccidioides* growth during 50/50 inhibition assay when in direct competition with *Coccidioides* on GYE agar *in vitro*. (**A**) Experimental setup: 50 *Coccidioides posadasii* Δ*cts2*/Δ*ard1*/Δcts3 arthroconidia were spread on half of a GYE agar plate, and intestinal microbiota or PBS/blank control was spread simultaneously on the other half of the plate. *Coccidioides* growth area was measured at days 4, 7, and 11. (**B**) *Coccidioides* grown against controls (PBS/blank) or (C) in direct competition with the intestinal microbiota. (**D**) Area of *Coccidioides* growth at measured time points. Circles represent mean and errors represent the standard deviation; blue, closed circle: control; red, open circle: intestine; *n* = 5–7. Statistical analysis was performed with two-way ANOVA; *P < 0.05, **P < 0.005.

Bacteria in the soil can exhibit an antagonistic effect on the growth of *Coccidioides in vitro* (17). To determine if host microbiota has the potential to inhibit *Coccidioides* growth, we placed *Coccidioides* and host microbiota in direct competition *in vitro*. We began inhibition assay experiments with small intestine microbiota because the intestine has a dense bacterial population that grows well *in vitro*. This method allowed us to survey a broad and unbiased range of culturable aerobic host microbiota. There were limitations in growing anaerobic bacteria to confluency and in culturing *Coccidioides* under anaerobic conditions; thus, anaerobic bacteria were not assessed in this study. By plating small intestinal microbiota and *Coccidioides* simultaneously on their respective halves of the agar plate, we provided an equal opportunity for the fungus and microbiota to compete for nutrients and space (Fig. 1A through C). *Coccidioides* growth area was measured at days 4, 7, and 11 post-spread on control plates and compared to microbiota experimental plates. Although day 4 was not statistically significant, inhibition of fungal growth was observed by day 4 in the presence of the small intestine microbiota (Fig. 1D). On days 7 and 11, *Coccidioides* growth area was significantly decreased when *Coccidioides* was placed in direct competition with the small intestine microbiota compared to controls. *Coccidioides* growth area averaged 45.5 cm^2^ on day 7 and 55.5 cm^2^ on day 11 in control plates, whereas *Coccidioides* area averaged 31.8 cm^2^ on day 7 and 45.3 cm^2^ on day 11 against intestine microbiota. Thus, the small intestine microbiota has an antagonistic effect, inhibiting *Coccidioides* growth by 31.8%, 30.2%, and 18.4% on days 4, 7, and 11, respectively.

**Fig 2:**
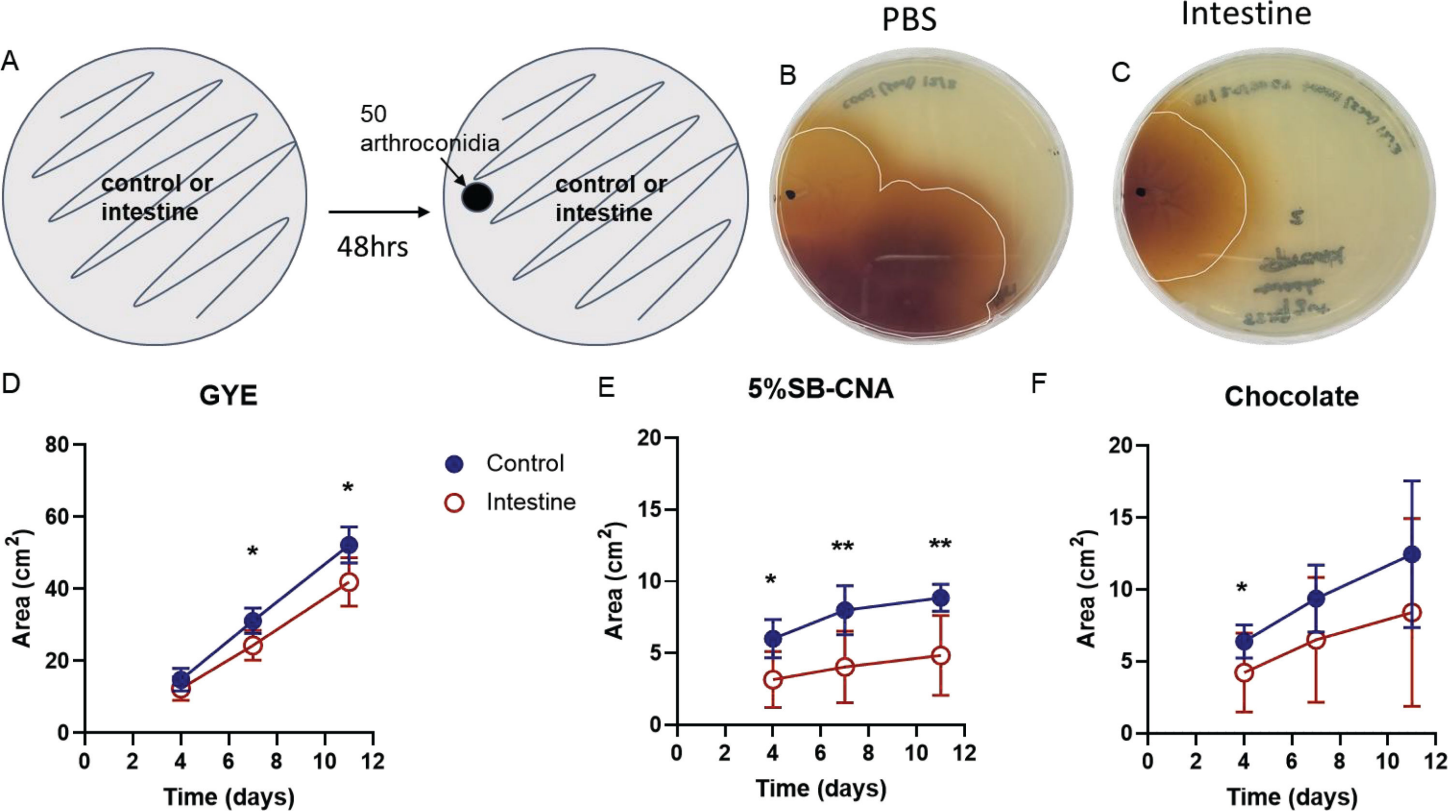
An established intestinal microbiota inhibits *Coccidioides* growth in *in vitro* spike-in inhibition assay. (**A**) Experimental setup: 50 *Coccidioides* arthroconidia were spiked onto a growing lawn of intestinal microbiota or control plate, and the area of *Coccidioides* growth was measured at days 4, 7, and 11. (**B**) *Coccidioides* spiked onto controls. (**C**) *Coccidioides* spiked on a ~80% confluently established lawn of intestinal microbiota. (**D–F**) *Coccidioides* growth area at measured time points. Circles represent the mean and errors represent the standard deviation; blue, closed circle: control; red, open circle: intestine on (D) GYE, (**E**) 5%SB-CNA, and (F) chocolate agar plates; *n* = 7–17. Statistical analysis was performed with mixed effect and two-way ANOVA; *P < 0.05, **P < 0.005.

While the direct inhibition assay served to assess the inhibitory potential of the host microbiota, we next sought to mimic the *in vivo* scenario in which the host microbiota is established prior to a *Coccidioides* infection. To achieve this, we allowed the small intestine microbiota growth to establish over 48 h prior to spiking in *Coccidioides* to mimic an infection. GYE is the optimal growth media for *Coccidioides,* whereas 5%SB-CNA primarily favors Gram-positive bacteria, and chocolate agar media favors Gram-negative fastidious bacteria, and these agars are used in clinical settings for diagnosis (29). Thus, we used these media types to favor the growth of *Coccidioides* or host microbiota, respectively, to observe the inhibitory potential in the presence of different nutrient sources. Small intestinal microbiota grown on GYE and 5%SB-CNA agar inhibited the growth of *Coccidioides*, which was depicted visually and numerically by a decreased area of fungal growth on small intestinal microbiota plates compared to controls (Fig. 2; Table S1). On GYE, *Coccidioides* grew to 31.2 cm^2^ on day 7 and 52.2 cm^2^ on day 11 in the controls as opposed to 24.3 cm^2^ on day 7 and 42 cm^2^ on day 11 when spiked onto an established lawn of small intestine microbiota (Fig. 2D). On 5%SB-CNA, *Coccidioides* grew to 8 cm^2^ on day 7 and 8.9 cm^2^ on day 11 in controls as opposed to 4 cm^2^ on day 7 and 4.8 cm^2^ on day 11 when spiked onto the established lawn of small intestine microbiota (Fig. 2E). The small intestine microbiota selected for growth by chocolate agar media did not significantly inhibit fungal growth (Fig. 2F). As expected, *Coccidioides* did not grow as well on 5%SB-CNA or chocolate agar compared to GYE; however, *Coccidioides* growth continued throughout the experiments.

**Fig 3:**
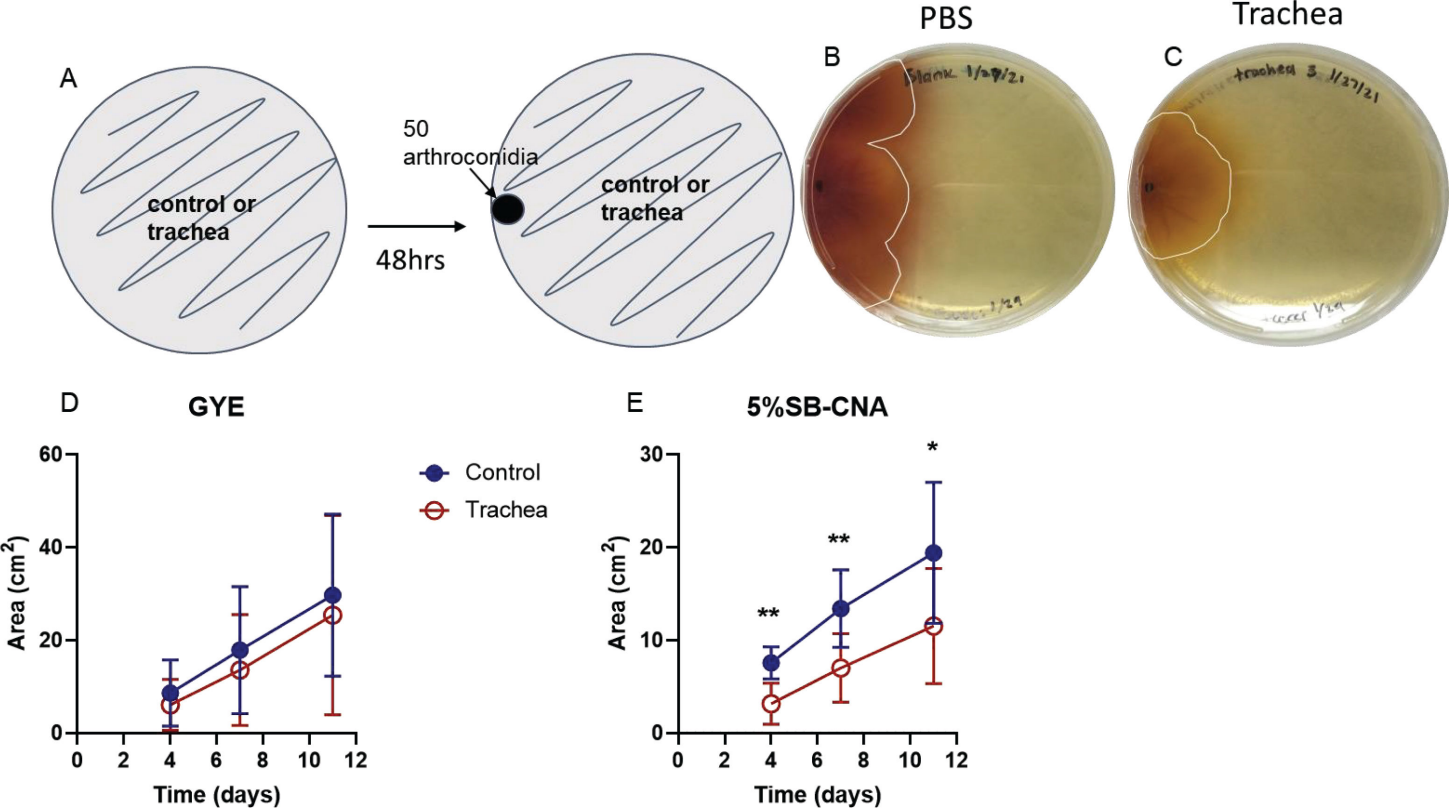
Tracheal mouse microbiota inhibits *Coccidioides* growth in *in vitro* spike-in inhibition assay. (**A**) Experimental setup: 50 *Coccidioides* arthroconidia were spiked onto a growing lawn of tracheal microbiota or control plate, and the area of *Coccidioides* growth was measured at days 4, 7, and 11. (**B**) *Coccidioides* spiked onto controls (1× PBS or blank). (**C**) *Coccidioides* spiked onto a ~80% confluent and established lawn of tracheal microbiota. (**D and E**) Area of *Coccidioides* growth at measured time points. Circles represent the mean and errors represent the standard deviation; blue, closed circle: control; red, open circle: trachea on GYE (D) and 5%SB-CNA (E) agar plates; *n* = 7–15. Statistical analysis was performed with two-way ANOVA; *P < 0.05, **P < 0.005.

Since an established lawn of small intestine microbiota inhibited *Coccidioides* growth, we next assessed the inhibitory potential of host microbiota cultured from a more relevant organ for *Coccidioides* infection. Although the lung is the primary site of *Coccidioides* infection, lung microbiota is notoriously difficult to culture (30, 31). Thus, we used the trachea as it is a part of the lower respiratory system and is indistinguishable from the upper respiratory system in healthy individuals (21). Tracheal microbiota can also be cultured directly by trachea spread onto agar plates. GYE or 5%SB-CNA agar plates were used to favor *Coccidioides* or host microbiota, respectively. Tracheal microbiota did not grow to confluency on chocolate agar plates; thus, these plates were not used. Tracheal microbiota cultured on 5%SB-CNA agar media displayed inhibitory potential on *Coccidioides* growth (Fig. 3; Table S2). This inhibition was depicted visually and numerically by the decreased fungal growth area on plates with tracheal microbiota compared to controls. On GYE, *Coccidioides* growth showed differences in several individual experiments (Fig. 3B and C) but was not statistically significant when the data were pooled (Fig. 3D), perhaps due to inconsistent growth or the low density of the inhibitory species. On 5%SB-CNA, *Coccidioides* grew to 7.7 cm^2^ on day 4 and 14 cm^2^ on day 7 in the controls as opposed to 3 cm^2^ on day 4 and 7.4 cm^2^ on day 7 when spiked onto an established lawn of tracheal microbiota (Fig. 3E; Table S2). Thus, *Coccidioides* growth was inhibited to some extent by tracheal microbiota grown on both types of media.

**Fig 4:**
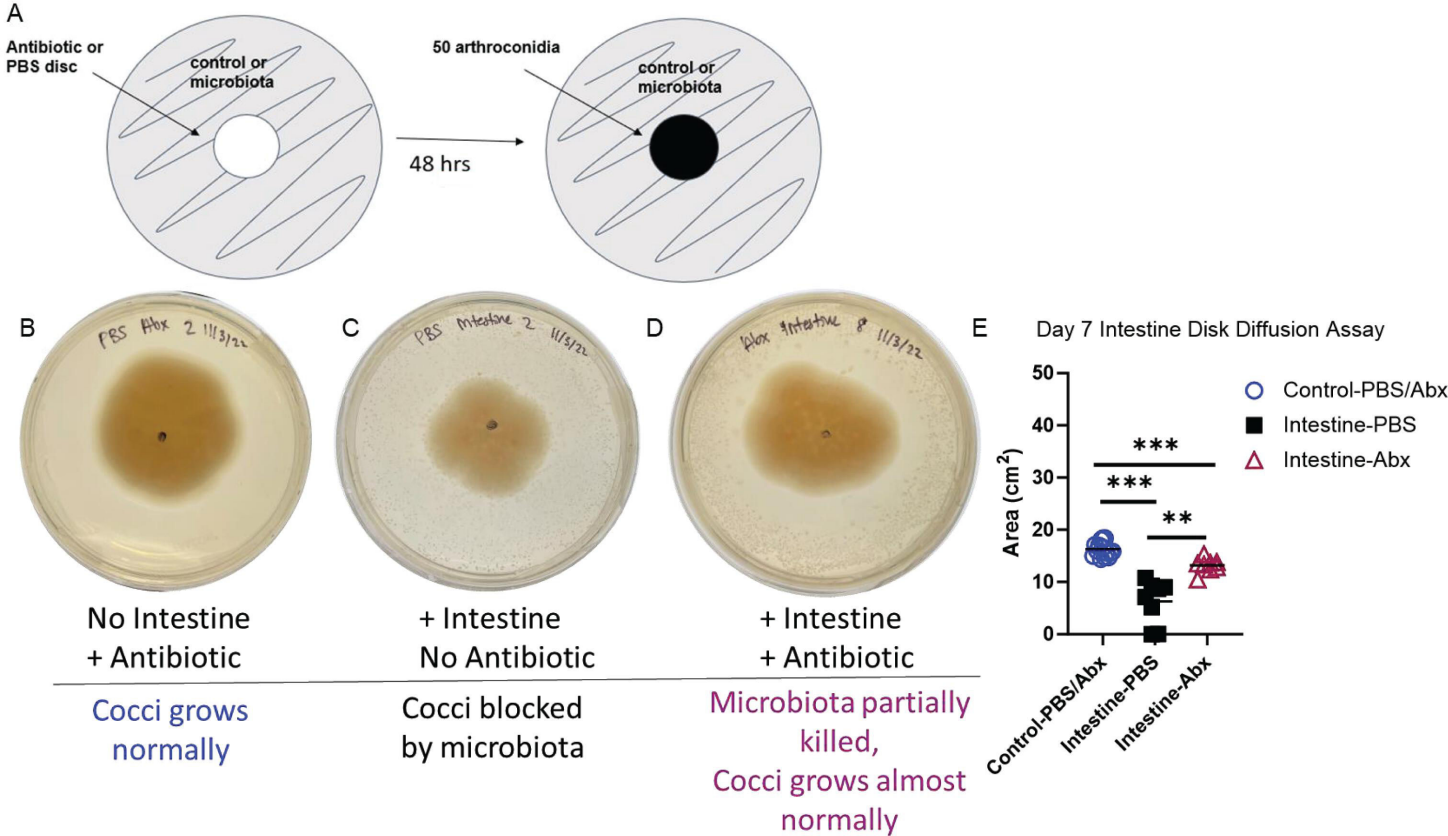
Antibiotic depletion of intestinal microbiota by antibiotic disk diffusion allows a niche for *Coccidioides* colonization and growth *in vitro*. (**A**) Experimental setup: microbiota or control was spread, and an antibiotic or PBS control disk was placed in the center of the plate for 48 h. Disks were removed at 48 h, and *Coccidioides* was spiked onto a growing microbiota lawn or control. *Coccidioides* growth area was measured at day 7. (**B**) Representative pictures of *Coccidioides* spiked onto controls or (C) onto a growing microbiota lawn treated with PBS disk or (D) onto a growing microbiota lawn treated with an antibiotic cocktail (ampicillin, rifampicin, streptomycin, and neomycin; 50 µg/mL each) disk. (**E**) Area of *Coccidioides* growth at day 7. Blue circles: control; black squares: intestine spread with PBS disk; purple triangle: intestine spread with antibiotic disk on GYE agar. *n* = 6–11. Statistical analysis was performed with unpaired parametric *t*-test with Welch’s correction; **P < 0.005, and ***P < 0.0005.

The Kirby-Bauer disk diffusion susceptibility test is typically used to determine the susceptibility of bacteria to an antimicrobial compound. Susceptibility is measured by the presence or absence of microbial growth around the disks. We used the disk diffusion assay to clear a zone of plated intestinal microbial growth using a cocktail of broad-spectrum antibiotics (ampicillin, rifampicin, streptomycin, and neomycin; 50 µg/mL each), mimicking antibiotic treatment *in vivo*. PBS disks did not disrupt the surrounding bacterial growth and were used as a control. Fungal growth was not disrupted on control plates treated with disks soaked in PBS or antibiotics (Fig. 4B). The area of growth between the two controls was not statistically significant; thus, these data were pooled. When comparing *Coccidioides* growth on PBS versus antibiotic-treated host microbiota plates, we observed that the area of growth was larger on antibiotic disk-treated plates than on plates treated with a PBS disk (Fig. 4C and D). Day 7 growth had the most pronounced differences with the area of *Coccidioides* growth being 6.25 cm^2^ on the host microbiota with the PBS disk versus 13.19 cm^2^ on the host microbiota with the antibiotic disks (Fig. 4E). Thus, the elimination of the intestinal microbiota with the use of the antibiotic cocktail created a niche for *Coccidioides* growth. Similar fungal inhibition results were observed on days 4 and 11 (data not shown), as shown on day 7. *Coccidioides* growth was inhibited when the microbiota was present, further confirming the potential of the microbiota to have an inhibitory effect on *Coccidioides*.

**Fig 5:**
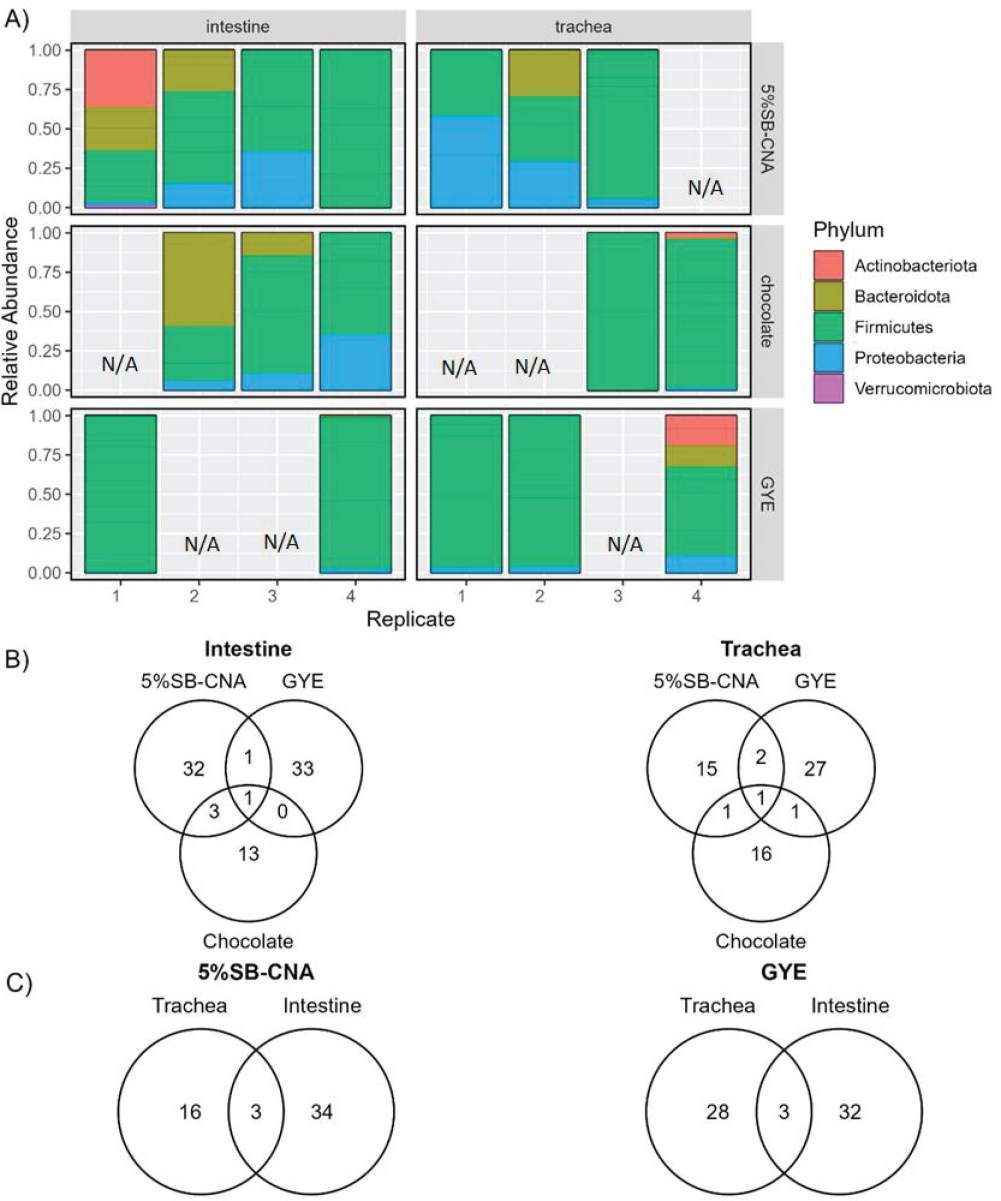
Bacterial composition and the relationships among tracheal and intestinal agar plates. (**A**) Relative abundance of ASVs at the phylum level in plated organ samples by plate type (row) and organ (column). (**B**) Venn diagram depicting the number of shared and unique ASVs among three plates (5%SB-CNA, GYE, and chocolate) for the plated intestine samples (left) and trachea plated samples (right), respectively. (**C**) Venn diagram showing the number of shared and unique ASVs between trachea and intestine for the samples plated on GYE plates (left) and 5%SB-CNA plates (right), respectively. N/A, missing replicates removed due to low/poor DNA.

To identify the bacteria responsible for the inhibition of *Coccidioides*, 16S rRNA sequencing of tracheal and intestinal growth on GYE, 5%SB-CNA, and chocolate plates was performed. The absolute abundance of ASVs at the phylum level varied between replicates of each organ on each agar type (Fig. S1). However, relative abundance ratios of ASVs at the phylum level were fairly consistent among organ and agar types (Fig. 5A). At the phylum level, the plated tracheal and intestinal growths were both primarily dominated by Firmicutes on all agar types and secondarily by Bacteroidota on 5%SB-CNA and chocolate agars (Fig. 5A). On GYE, Proteobacteria was found on all trachea and intestinal replicates (Fig. 5A). At the family level, plated tracheal growths were primarily dominated by *Staphylococcaceae* on all agar types, while plated intestinal growths were primarily dominated by *Lactobacillaceae* on GYE agar and *Staphylococcaceae* on chocolate agar (Fig. S2). 5%SB-CNA plates were dominated by different families among replicates (Fig. S2). Although the trachea and intestine are rather distinct in their environmental conditions and composition, bacterial composition was similar at the phylum level. This similarity diminished at the lower taxonomic levels, highlighting unique ASVs among the three agar plates (Fig. 5B), although common ASVs did remain at the genus level. Bacteria from the *Staphylococcus* genus were shared between tracheal growths on GYE and 5%SB-CNA agar types, which culture bacteria with inhibitory potential against *Coccidioides* in spike-in assays (Fig. 5B; Table 1). One ASV from the family *Lactobacillaceae* was uniquely shared by the two agar types of interest, 5%SB-CNA and GYE, in the intestine (Fig. 5B; Table 1). Since both the plated tracheal and intestinal growth showed inhibitory potential, we next sought to identify shared ASVs between the trachea and intestine samples plated on GYE plates and 5%SB-CNA plates, respectively (Fig. 5C). Tracheal and intestinal growths on GYE shared three ASVs, as did growths on 5%SB-CNA (Fig. 5C). On GYE plates, all three ASVs were of the *Lactobacillus* genus, while the 5%SB-CNA plates included ASVs from *Mitochondria*, *Lactobacillaceae*, and *Staphylococcaceae* families (Table 2). Comparing bacterial growth on agar types with the inhibition of *Coccidioides* allowed further characterization of bacteria with inhibitory potential for future study.

**TABLE 1:** ASVs identified in the intestine or trachea samples when grown on 5%SB-CNA and GYE plates but not on chocolate agar^*a*^

Organ	Plate	Phylum	Family	Genus
Intestine	$B\cap G\cap C^{c}$	Firmicutes	*Lactobacillaceae*	NA
Trachea	$B\cap G\cap C^{c}$	Firmicutes	*Staphylococcaceae*	*Staphylococcus*
		Firmicutes	*Staphylococcaceae*	*Staphylococcus*

^*a*^ B, 5%SB-CNA; G, GYE; C, chocolate; c, excluding; ∩, shared; and NA, could not be identified.

**TABLE 2:** Information of ASVs present in both the trachea and intestine samples plated on GYE and 5%SB-CNA plates, respectively^*a*^

Plate	Phylum	Family	Genus
GYE	Firmicutes	*Lactobacillaceae*	*Lactobacillus*
	Firmicutes	*Lactobacillaceae*	*Lactobacillus*
	Firmicutes	*Lactobacillaceae*	*Lactobacillus*
5%SB-CNA	Proteobacteria	*Mitochondria*	NA
	Firmicutes	*Lactobacillaceae*	NA
	Firmicutes	*Staphylococcaceae*	*Staphylococcus*

^*a*^ NA, could not be identified.

**Fig 6:**
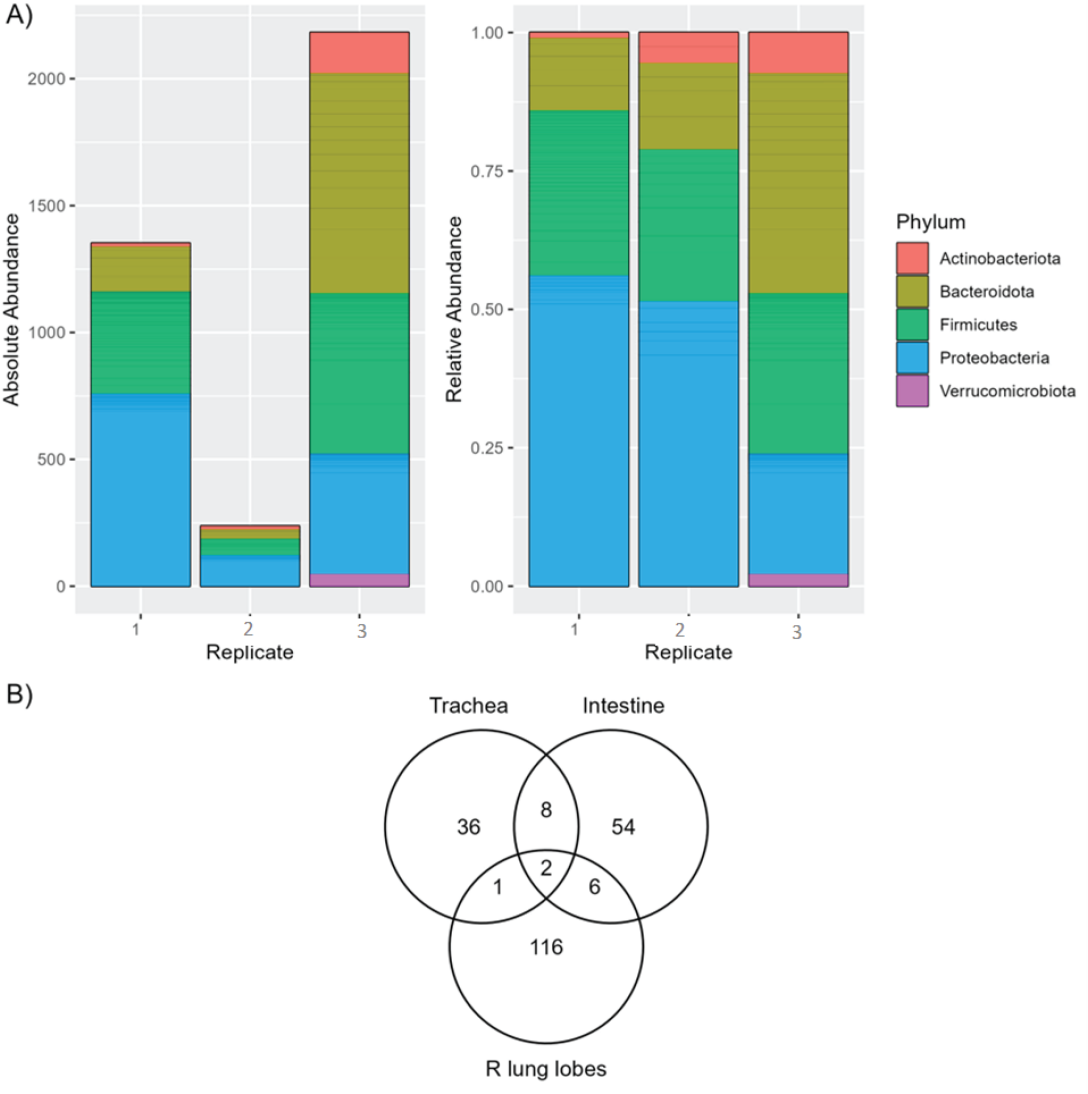
Bacterial composition of the right lung lobe. (**A**) Phylum-level comparison of ASV absolute abundance (left) and relative abundance (right) in whole organ right lung lobe samples. (**B**) Venn diagram showing the number of shared and unique ASVs between trachea and intestine for the samples on GYE and 5%SB-CNA plates and the whole right lung lobes. R, right

As the lung microbiota is refractory to *in vitro* culture, mouse lung extracts were sequenced and compared to cultured intestine and trachea for overlapping bacterial identification. The lung microbiota was nearly evenly dominated by Proteobacteria, Firmicutes, and Bacteroidota in order of relative abundance at the phylum level (Fig. 6A). Multiple ASVs were shared among the plated organs and right lung extracts. The trachea, lung, and intestine shared two ASVs, *Muribaculaceae* and *Bifidobacteriaceae* at the family level (Fig. 6B). The trachea and lung shared *Achromobacter* at the genus level, which is not present in the intestine. Finally, the trachea and intestine predominantly shared bacteria in the *Lactobacillaceae* family, which were not present in the lung (Fig. 6B).

## DISCUSSION

The presence of host microbiota derived from either the intestine or trachea inhibits the growth of *Coccidioides* arthroconidia *in vitro*. We focused on the interactions at the arthroconidia stage as it is the first stage to interact with the microbiome and we sought to identify if the microbiome has the same inhibition potential observed by soil bacteria. In the host, *Coccidioides* undergoes a phase change and persists as spherules. Microbiome inhibitory capacity against *Coccidioides* spherules was not assessed in this study. Nonetheless, the intestinal microbiota inhibits *Coccidioides* arthroconidia growth both when they are placed in direct competition and when the host microbiota is allowed to establish first. Regardless of whether culture conditions provide an equal opportunity for the host microbiota and *Coccidioides* to compete or mimic an *in vivo* scenario in which we allow the microbiota to establish prior to infecting with *Coccidioides*, *Coccidioides* growth is inhibited. The tracheal microbiome is less dense in bacterial composition than the small intestine; thus, not all plates spread with tracheal microbiota reached confluency. Therefore, only spike-in inhibition assays were performed, and only tracheal growths that reached ~80% confluency were utilized. In these assays, tracheal microbiota also inhibited *Coccidioides* growth. There were differences observed in the level of inhibition based on the type of agar used. Intestinal microbiota cultured on 5%SB-CNA agar display inhibitory effects, whereas microbiota cultured on chocolate agar do not. The differences demonstrate that it is not simply the presence of microbiota that is responsible for inhibition, but rather the different types of microbes selected by nutrients in the media type. 5%SB-CNA agar primarily selects for Gram-positive bacteria, whereas chocolate agar primarily selects for Gram-negative fastidious bacteria but is relatively nonselective. *B. subtilis-*like species, a Gram-positive bacteria species prevalent in the soil, displays antifungal activity against *Coccidioides in vitro* (17). Thus, the bacteria identified in 5%SB-CNA agar should be considered for antifungal activity. Other studies have found inconsistent success with growing *Coccidioides* under anaerobic conditions (32–35). We were unable to culture *Coccidioides* anaerobically on GYE (data not shown) and could not assess whether anaerobic microbiota have the capacity to inhibit *Coccidioides* growth. Using additional agar nutrients could help define host bacteria with the ability to inhibit *Coccidioides*, but *Coccidioides* exhibits limited growth when cultivated on Luria broth, Sabouraud dextrose with chloramphenicol, and MacConkey’s agars. This study uses the avirulent Δ*cts2*/Δ*ard1*/Δ*cts3 Coccidioides posadasii* strain that is missing the chitinase 2 and 3 genes. These genes are responsible for the digestion of the spherule septal wall complex to begin endospore differentiation. This avirulent strain has no known defects in mycelia and arthroconidia formation, but rather attenuation at the endosporulation stage (27). We predict that virulent *Coccidioides* would yield similar results *in vitro* as those observed in this study with avirulent arthroconidia.

Due to misdiagnosis, 60%–80% of Valley fever patients are treated with antibiotics (3). To determine how perturbing an established microbiome would affect the inhibitory potential of the microbiota on *Coccidioides*, we depleted the microbiota with an antibiotic cocktail *in vitro*. Depletion of host microbiota through an antibiotic disk allowed a niche for *Coccidioides* growth. Although these are not *in vivo* studies, the *in vitro* data presented demonstrate the potential consequences of improper antibiotic treatment from misdiagnosing Valley fever patients with bacterial pneumonia. Antibiotics may change the course of infection by altering the host microbiota and immune response (22, 36). Antibiotic treatment can cause proximal changes in the microbial composition of the intestine, which can lead to distal immunological changes in response to pulmonary infections. Antibiotics can also cause distal changes in the microbial composition of the lung. The *in vitro* data presented here demonstrate a direct influence of respiratory tract microbiota on *Coccidioides* growth and the effects that broad-spectrum antibiotic use has on inhibition capabilities.

Although the lungs are the primary site of *Coccidioides* infection, the trachea is also part of the lower respiratory system, and the intestine has proven to have an influence on respiratory infections through the gut-lung axis (23, 37, 38). Thus, we sequenced bacterial growth from the trachea and intestine on the three agar types to identify levels of taxonomic order unique to the agar types that enabled the growth of bacteria with inhibitory activity in our spike-in assays. Cross-comparison of the bacteria identified on each agar type and from each organ revealed shared ASVs among 5%SB-CNA and GYE agars, as well as those shared between the trachea and intestine samples plated on GYE and 5%SB-CNA. This allowed us to narrow down the candidates with potential inhibitory potential for future studies. To bring relevance to the pathogenesis of pulmonary *Coccidioides*, the right lung lobes of mice were sequenced for bacterial identification. As opposed to the plated cultures of tracheal and intestinal data, the lung data are from non-cultured whole lobe extracts due to the lung microbiota being notoriously difficult to culture (30, 31). To avoid extensive manipulation of the resident microbiota, the whole lung was processed for extraction without liquid culturing. We identified *Muribaculaceae* and *Bifidobacteriaceae* to be shared among all three organs and *Alcaligenaceae* to be shared between the trachea and right lung lobes (upper respiratory system) for future evaluation (Table 3).

**TABLE 3:** Information of ASVs present in the trachea and intestine samples, plated on 5%SB-CNA and GYE plates, and whole organ right lung lobe samples^*a*^

Organ	Phylum	Family	Genus	Note
T∩I∩L	Bacteroidota	*Muribaculaceae*	NA	*
	Actinobacteriota	*Bifidobacteriaceae*	*Bifidobacterium*	*
$T\cap I^{C}\cap L$	Proteobacteria	*Alcaligenaceae*	*Achromobacter*	
$T\cap I\cap L^{C}$	Firmicutes	*Lactobacillaceae*	*Lactobacillus*	
	Firmicutes	*Lactobacillaceae*	*Lactobacillus*	
	Proteobacteria	*Mitochondria*	NA	
	Firmicutes	*Lactobacillaceae*	NA	
	Firmicutes	*Staphylococcaceae*	*Staphylococcus*	
	Firmicutes	*Lactobacillaceae*	*Lactobacillus*	
	Firmicutes	*Lactobacillaceae*	NA	*
	Firmicutes	*Lactobacillaceae*	NA	*

^*a*^ Note that there are four ASVs shared between intestine samples plated on 5%SB-CNA plates and trachea samples plated on GYE plates, which are distinct from the ASVs shown in Table 2, and they are denoted with * in the table. T, trachea; I, intestine; L, lung; c, excluding; ∩, shared; and NA, could not be identified.

*Lactobacillus* and *Staphylococcus* were the predominant genera found in our plated sequencing data, likely because of the abundance and ease of culturing of these microorganisms. Previous work demonstrated that the oropharyngeal, lung, and gut microbiota of healthy mice are dominated by *Lactobacillus* species (39). However, it is possible that microorganisms that have inhibitory potential on *Coccidioides* growth exist within the host microbiota but are difficult or virtually impossible to culture *in vitro*. Although this is a limitation of our study, *Lactobacillus* species have shown antifungal effects *in vitro,* have been used as probiotics in viral and bacterial respiratory infection studies, and have improved infection outcomes (40–48). Cell-free supernatants of *Lactobacillus plantarum* UM55 and *Lactobacillus buchneri* UTAD104 were tested against the fungal contaminant *Penicillium nordicum* and a reduction of radial growth and production of ochratoxin A were observed (49). Acetic acid, indole lactic acid, and phenyllactic acid were the most effective in inhibiting *P. nordicum* growth and ochratoxin A^50^. *In vivo*, antibiotic-induced dysbiosis during upper respiratory tract infection with influenza A virus is restored by *Lactobacillus paracasei* 431 and *Lactobacillus fermentum* PCC (41). *Lactobacillus* strains restores the imbalance in the upper respiratory tract microbiome and re-upregulates pro-inflammatory cytokines (41). Mice treated with heat-killed *Lactobacillus gasseri* TMC0356 are protected against influenza virus infection by stimulating protective immune responses (42). On the other hand, few *Staphylococcus* species have been used as probiotics for therapeutic treatment as most are opportunistic pathogens that cause disease. *Staphylococcus aureus* colonizes the nose, and *Staphylococcus saprophyticus* colonizes the urinary tract. However, *Staphylococcus epidermidis* (*S. epidermidis*) has probiotic potential in multiple human and animal model studies. *S. epidermidis* can ameliorate infection by *Staphylococcus aureus*, *Moraxella catarrhalis*, Group A *Streptococcus*, influenza virus A, *Streptococcus pneumoniae*, and *Klebsiella pneumoniae* (50–55). Treating mice with *S. epidermidis* NRS122 and streptomycin reduces colonization by *Staphylococcus aureus* BD02-31 compared to mice that receive streptomycin alone (51). In a mouse model of influenza A, intranasally pre-colonizing with *S. epidermidis* limits the spread of influenza virus A to the lungs by modulating IFN-γ-dependent innate immune mechanisms (55). Yayurea A and B, small compounds isolated from *Staphylococcus delphini*, are expressed in a *Staphylococcus* species group (56). These compounds have inhibitory potential against Gram-negative bacteria (56). Additionally, bacteriocins proteins produced by *S. epidermidis* inhibit *Micrococcus luteus*, *Corynebacterium pseudodiphteriticum*, *Dolosigranulum pigrum*, and *Moraxella catarrhalis*, bacterial species frequently found in human nasal microbiomes (57). *S. epidermidis* bacteriocins might also be used against pathogenic bacteria. In addition to *S. epidermidis, Staphylococcus xylosus* VITURAJ10 also suppresses the growth of pathogenic strains of *Escherichia coli*, *Salmonella enterica*, and *Staphylococcus aureus* (58). *Staphylococcus succinus* AAS2 also displays antagonistic traits against *Staphylococcus aureus* (59). These studies with other respiratory infections are evidence for the potential of *Staphylococcus* species to be used as probiotic treatment to improve infection outcomes.

Sequencing data from the lung as well as tracheal and intestinal plates reveal that *Bifidobacterium* is shared among the three organs (Table 3). Randomized, controlled human clinical trials and mouse models have proven the efficacy of using *Bifidobacterium* as a probiotic during respiratory tract infections like *Klebsiella pneumoniae*, influenza, and rhinovirus infection (60–66). Oral treatment with commensal probiotic *Bifidobacterium longum* 5(1A) protects mice against *Klebsiella pneumoniae* pulmonary infection by activating Toll-like receptor signaling pathways that alter inflammatory immune responses (61). A randomized controlled study also revealed that *Bifidobacterium animalis* subspecies lactis BI-04 affects the baseline of innate immunity in the nose (63). Administering a single probiotic results in amelioration of many pulmonary infections; however, probiotic cocktails have also proven effective. Administering *Lactobacillus rhamnosus* GG in combination with *B. longum* improves lung injury following experimental infection (67). Thus, the bacteria with inhibitory potential against *Coccidioides* could be evaluated as probiotics alone or in combination for therapeutic treatment of coccidioidomycosis. This could provide a supplemental or alternative therapeutic to the existing antifungal therapies; however, further assessment would be necessary prior to implementation.

Among healthy individuals, the upper and lower respiratory tracts appear indistinguishable (21). However, the microbiota differs between the upper and lower respiratory tracts and even within the lungs among individuals with asthma, chronic obstructive pulmonary disease, and cystic fibrosis (19, 68–70). Recent studies on humans, macaques, and mice revealed that viral and bacterial infections cause shifts in the landscape of lung microbiota (24, 71–74). It is unknown whether *Coccidioides* infection causes microbiome shifts or how infection plus antibiotic treatment alters the lung microbiome. Our data suggest that an altered microbiome through antibiotic treatment may allow a niche for fungal growth. This is an area of study that requires further investigation in order to advise clinicians on the risks associated with antibiotic treatment during *Coccidioides* infection. Such findings could revolutionize the way infectious diseases are treated by leveraging microbiome interactions and probiotic therapeutics. Existing antifungal therapies for chronic and severe *Coccidioides* have unpleasant and severe side effects; exploring alternative treatments could improve patient outcomes and contribute significantly to our understanding of host-*Coccidioides* interactions.

## Data Availability

The raw data of 16S rRNA sequencing were deposited in the Sequence Read Archive (SRA) at NCBI under BiopProject PRJNA1081448.

## SUPPLEMENTAL MATERIAL

Supplemental material spectrum.02978-23-s0001.docx Tables S1 and S2; Figures S1 and S2.

ASM does not own the copyrights to Supplemental Material that may be linked to, or accessed through, an article. The authors have granted ASM a non-exclusive, world-wide license to publish the Supplemental Material files. Please contact the corresponding author directly for reuse.
