# Supplementary material for: Host tracheal and intestinal microbiomes inhibit Coccidioides growth in vitro
Source: Microbiol Spectr. 2024 Jun 4;12(7):e02978-23. doi: 10.1128/spectrum.02978-23 (PMC11218535; doi:10.1128/spectrum.02978-23)
Supplement: Supplemental material — Tables S1 and S2; Figures S1 and S2. [file spectrum.02978-23-s0001.docx]

|  | Day 4 | | | | Day 7 | | | | Day 11 | | | |
| --- | --- | --- | --- | --- | --- | --- | --- | --- | --- | --- | --- | --- |
|  | Control  (cm^2^) | Experimental  (cm^2^) | %Inhibition | p-value | Control  (cm^2^) | Experimental  (cm^2^) | %Inhibition | p-value | Control  (cm^2^) | Experimental  (cm^2^) | %Inhibition | p-value |
| GYE | 14.84 | 12.38 | 16.6% | 0.3888 | 31.18 | 24.3 | 21.9% | 0.0103 | 52.2 | 42.0 | 19.6% | 0.0387 |
| 5%SB-CNA | 6.0 | 3.2 | 47.5% | 0.00114 | 8.0 | 4.0 | 49.5% | 0.0065 | 8.9 | 4.8 | 45.4% | 0.0066 |
| Chocolate | 6.4 | 4.2 | 34.1% | 0.0217 | 9.37 | 6.5 | 30.6% | 0.0810 | 12.5 | 8.5 | 32% | 0.1759 |

**Supplemental Table 1: Intestinal spike in % inhibition**

|  | Day 4 | | | | Day 7 | | | | Day 11 | | | |
| --- | --- | --- | --- | --- | --- | --- | --- | --- | --- | --- | --- | --- |
|  | Control  (cm^2^) | Experimental  (cm^2^) | %Inhibition | p-value | Control  (cm^2^) | Experimental  (cm^2^) | %Inhibition | p-value | Control  (cm^2^) | Experimental  (cm^2^) | %Inhibition | p-value |
| GYE | 8.0 | 6.1 | 24.1% | 0.7734 | 17.9 | 13.6 | 24.1% | 0.8540 | 29.7 | 25.4 | 14.5% | 0.9539 |
| 5%SB-CNA | 7.7 | 3.0 | 61.3% | 0.0012 | 14.0 | 7.4 | 47.2% | 0.0051 | 20.1 | 12.0 | 40.3% | 0.0472 |

**Supplemental Table 2: Tracheal spike in % inhibition**


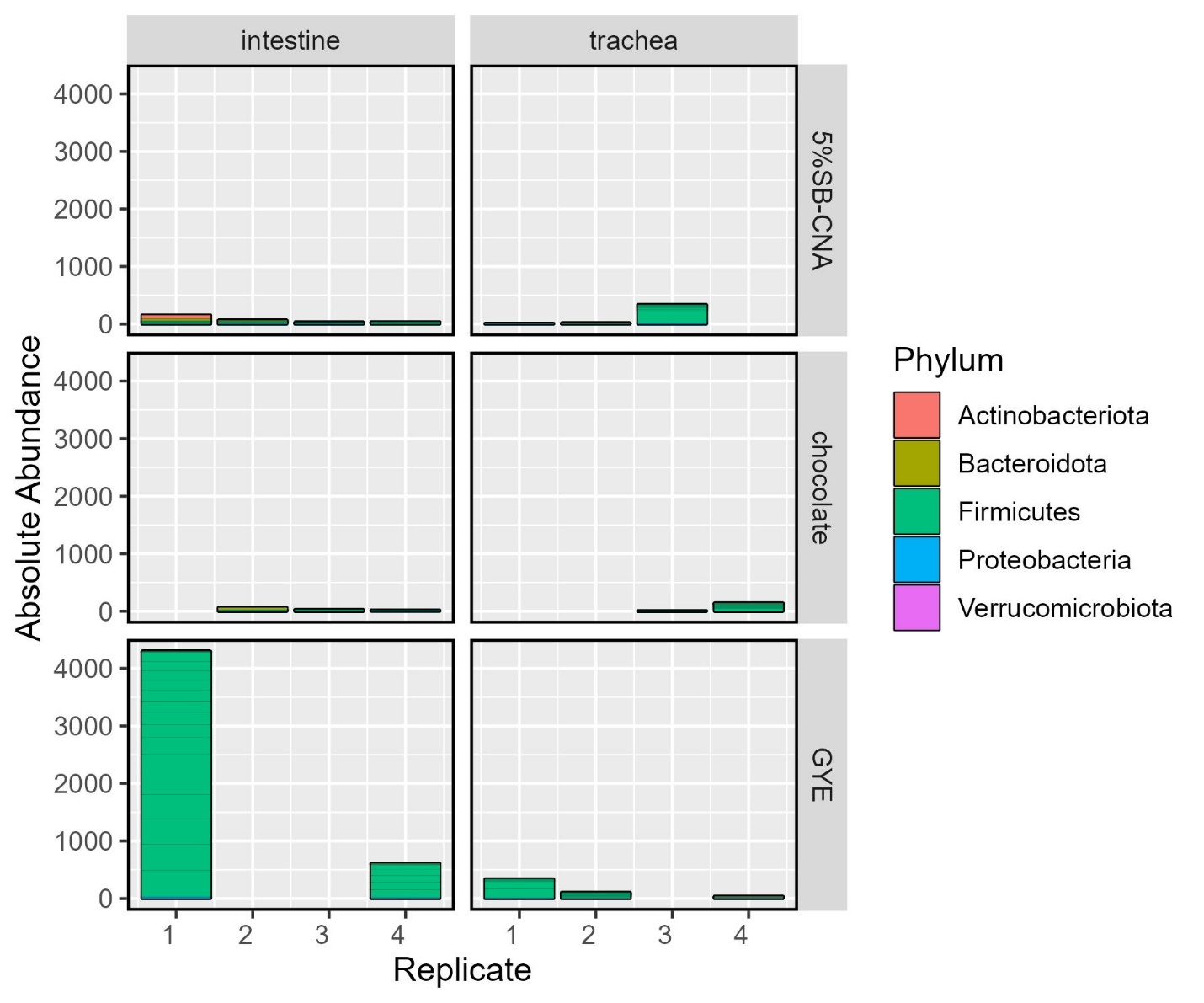


N/A

N/A

N/A

N/A

N/A

N/A

N/A

Supplemental Figure 1: Phylum-level comparison of ASV absolute abundance in plated organ samples by plate type (row) and organ (column). Note: NA= missing replicates removed due to low/poor DNA.


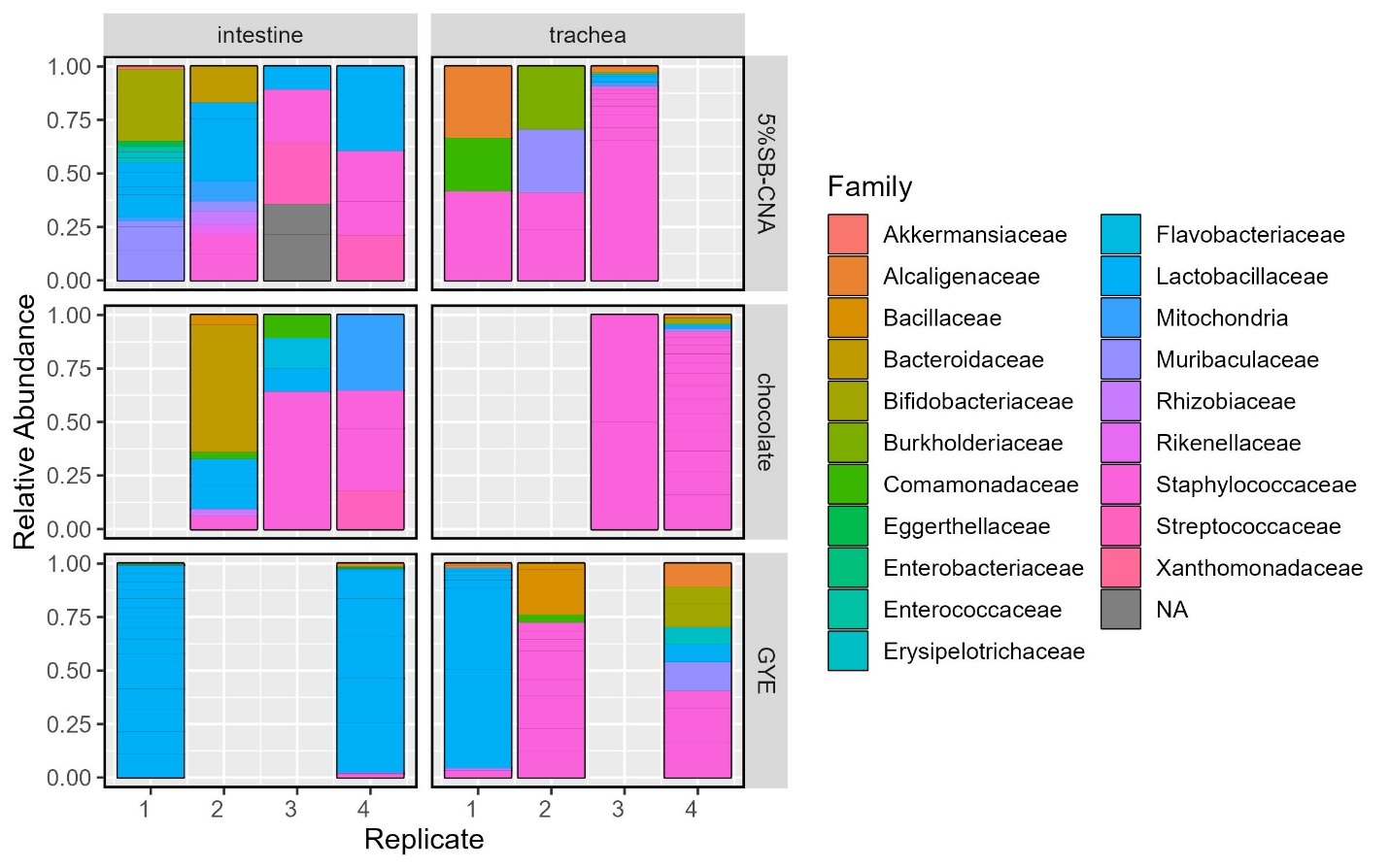


NI

N/A

N/A

N/A

N/A

N/A

N/A

N/A

Supplemental Figure 2: Family-level comparison of ASV relative abundance in plated organ samples by plate type (row) and organ (column). Note: NI= could not be identified, N/A= missing replicates removed due to low/poor DNA.
